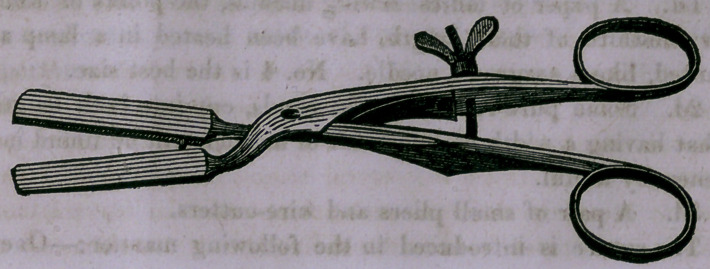# A Case of Complete Prolapsus of the Rectum, Operated upon by “Smith’s Method”

**Published:** 1867

**Authors:** J. W. Freer

**Affiliations:** Prof. Physiology and Surgical Pathology, Rush Medical College, Chicago, Ill.


### CHICAGO MEDICAL JOURNAL.

Vol. XXIV. APRIL, 1867. No. 4.

### ORIGINAL CONTRIBUTIONS.

*A Case of Complete Prolapsus of the Rectum, Operated upon by "Smith's Method."* By J. W. Freer, M.D., Prof. Physiology and Surgical Pathology, Rush Medical College, Chicago, Ill.

Case.—Andrew S., *æt.* 32, American, presented himself in the fore part of April, 1866, under the following conditions, *viz.*:—a complete prolapsus of the rectum, which had existed, according to his statement, about twenty years, or since his recollection. On placing himself in a sitting posture, he was able to expel what seemed to be the entire rectum, leaving no sulcus between the margin of the anus and mucous membrane. The size of the prolapsed gut was 10 inches long and 20 inches in circumference, by measurement, around the middle. The mucous membrane, from long exposure, had taken on the cuticular formation—no ulceration existed, nor was there unusual vascularity.

The bowels always protruded at the time of defecation, and had to be restored by the hand, followed, for a space of time, by intense tenesmus, pain in the back, and irritability of the bladder. These painful symptoms were such that the evacuations were usually deferred until the last moment, (sometimes

10

"going a week or ten days without a passage,") and then had to be induced by cathartics, castor-oil being his favorite remedy. Finally, his condition was such as to totally incapacitate him for the ordinary affairs of life.

My first impulse was to advise the man to go home and bear his ills as best he could, for I could not recall either authority or precedent for any operation which might promise benefit, but that of Dupuytren's—by removing V shaped folds from the verge of the anus, and this in the almost total absence of the sphincter muscle, and in view of the enormous size of the prolapsus seemed quite hopeless. However, at the urgent solicitation of the patient, I finally consented to perform *some* operation, having not, as yet, fully determined in my own mind the method to be adopted. Subsequently, I called into consultation my friend Dr. A. J. Baxter, who has had considerable experience in the use of Smith's clamp in the treatment of internal hemorrhoids—he being the first to introduce it into this city, some two years ago. The result of the conference was, that of adopting the following operation, *viz.:*—removing by the clamp and actual cautery a series of longitudinal folds extending from base to apex of the tumor.

*Operation.*—April 30th, with the assistance of Drs. Baxter, Hunt, and Cole, the operation was performed as follows:—The patient being placed semi-prone, the tumor fully protruded, and chloroform administered to a degree of anæsthesia, I removed vertical folds, extending the entire length of the tumor, to the number of nine, equally distributed around the circumference of the cylinder. No untoward symptoms followed; the sloughs separated on the third day, and were evacuated without fæcal matter. On the fourth, a castor-oil emulsion was given, which caused a free movement, without pain or protrusion of the bowel, a circumstance which, he remarked, had not occurred for years. On the fifth day, he was going about the hotel, and on the seventh departed for home, by rail, a distance of 120 miles.

From this time to the middle of September, there had been no return of his infirmity. In his communications he averred that his health was restored, and, to quote his own language,

"I consider myself cured, and have resumed my avocation"—that of farming. Unfortunately, about this time, he was seized with acute dysentery, which resulted in a partial return of his former trouble. On the 28th of November, he again presented himself for another operation, which, I may briefly state, was a repetition of the first, with the exceptions of removing some pendulous folds from the verge of the anus, and also two transverse folds intersecting the longitudinal at right angles.

In the first and last operations, there were some variations from Smith's method of removing, with scissors or bistoury, the included folds of mucous membrane before applying the cautery. Finding, after removing two folds by the above method, that some hemorrhage followed, I subsequently applied the cautery at once, leaving the eschar untouched. With this modification, no bleeding followed. The cautery was applied so thoroughly that the tissues were completely charred.

*Present Condition.*—March 4th, I received a letter, from which I quote the following paragraph:—"I am enjoying excellent health, but it comes down a little on one side. If it gets any worse I will come in and have some more taken off."

*Remarks.*—So far as I have been able to ascertain, the case above related is the first of like magnitude that has been subjected to similar treatment, and, to the best of my knowledge, in cases of this kind, we have no authority for any other operation than the removal of V shaped folds from the margin of the anus, but in severe cases, like the above, where all the tunics are involved in a state of extreme laxity and mobility, the sphincter muscle atrophied and nearly or quite functionless, this operation, as shown by experience, usually terminates either in failure to give relief, or, if too much tissue have been removed, in one of the most deplorable of all conditions—stricture of the rectum.

We do not propose to discuss the comparative merits of the clamp, ligature, and nitric acid in internal hemorrhoids and trifling cases of prolapsus recti, these, as far as treatment may be concerned, having the least possible relation to conditions like the above. In these minor affections, either method is sufficiently successful—favorable results almost uniformly following. But where extensive ablation of mucous membrane is required, we believe the clamp possesses the following advantages, *viz.*:—facility of execution, risk from hemorrhage avoided, and more speedy recovery—the time consumed not generally exceeding five to eight days.

Ashton, in his invaluable work on the Rectum, says:—"in some cases, on account of age, debility, or *other circumstances*, an operation cannot be performed." Now, in his report of cases treated by himself, no mention is made of any other (comparatively speaking) than minor cases. Therefore, may we not safely conclude that he refers, in the above quotation, as much to certain local circumstances of the prolapse, such as volume, relaxed condition of the pelvic floor, etc., as to general conditions of the constitution.

Gross, in his work on Surgery, speaks of a remarkable case of prolapse in the person of an adult Mississippian. He says: "the disease had troubled him for a long time, and the tumor was fully as large as the crown of an ordinary-sized hat." He omits to make mention of the treatment or final result. In a general way, he recommends as treatment (in severe cases), "the excision of some of the cutaneous folds of the ano-gluteal region." By this operation, he says, "contraction of the anal orifice is *hoped* for, and will rarely disappoint expectation." Again, he speaks of having helped "Prof. Richardson in such an operation, but although it was well executed, no appreciable benefit resulted. The patient was a middle-aged woman, who had for years labored under an immense prolapsus of the lower gut, attended with great and permanent relaxation of the integuments and muscles of the anus, which resisted every mode of treatment that could be devised."

Mr. Henry Smith says, in his article on Prolapsus, *Holmes' Surg.*, vol iv., p. 197:—"The object to be obtained is, to reduce the redundancy or relaxation of the mucous membrane, to promote adhesion between the several tissues composing the bowel, and to brace up the anus and sphincter." In his treatment "for severe cases"—notwithstanding he is the hero of the method by "the improved clamp"—he recommends Hay's operation, as modified by Dupuytren, or the ligature, respecting which, he says:—"another mode of curing prolapsus, consists in the application of the ligature to portions of the prolapsed membrane. This plan is especially adapted to those cases where there is great laxity of the mucous membrane, and where the surrounding integument is not much involved," but, as far as I know, he has never reported cases treated in this manner, where more than simple segments of the gut were involved; we are, therefore, warranted in the conclusion that he does not refer to cases like the one embraced in this report, for, certainly, it would be a formidable proceeding to include in ligatures a superficies of mucous membrane equal in extent "to an ordinary hat crown."

Such, I believe, is a fair representation of the literature on this subject, up to the present time, and if we have, in Smith's improved clamp, a safe and effectual means of attacking these "severe cases," may we not cangratulate ourselves upon having the means of removing another of the *opprobria* from the fair escutcheon of surgical science and art.

The favorable termination, we venture to suggest, was due to two principal causes, *viz.*:—the reduction of the size of the prolapsus by the removal of tissue, and the induction of an extensive adhesive inflammation as a result of the cauterization, the latter, doubtless, playing a more important part in the case than the former, and this by the production of new connective tissue between the intestines and surrounding parts, in a manner inducing nature to replace the long-lost natural supports of the organ.

NOTE.—A sketch of the instrument used in the foregoing operation by Prof. Freer, was given in a previous volume. For the benefit of new subscribers, it is here reproduced. It so nearly explains itself, that very little description is needed. A somewhat similar clamp was long ago recommended by Mr. Curling, but Smith's differs from it by having the opposed surfaces of the blades fitting to each other by a groove and mortice, or by respective concavity and convexity, also by the use of the screw instead of a catch in the handle. These, which would appear mere modifications, practically are as diverse in application as a pair of scissors from a dentist's forceps. Mr. Smith's gives a perfect adaptation and control of the parts which cannot be secured, save accidentally, by the use of Mr. Curling's instrument. [EDITOR.

## Figures and Tables

**Figure f1:**